# Use of deep learning in the MRI diagnosis of Chiari malformation type I

**DOI:** 10.1007/s00234-022-02921-0

**Published:** 2022-02-24

**Authors:** Kaishin W. Tanaka, Carlo Russo, Sidong Liu, Marcus A. Stoodley, Antonio Di Ieva

**Affiliations:** 1 Macquarie Medical School, Macquarie University, NSW 2109 Sydney, Australia; 2 Computational NeuroSurgery (CNS) Lab, Macquarie University, Sydney, Australia; 3 Centre for Health Informatics, Australian Institute of Health Innovation, Faculty of Medicine, Health and Human Sciences, Macquarie University, Sydney, Australia

**Keywords:** Artificial intelligence, Chiari I malformation, Convolutional neural network, Deep learning, Magnetic resonance imaging

## Abstract

**Purpose:**

To train deep learning convolutional neural network (CNN) models for classification of clinically significant Chiari malformation type I (CM1) on MRI to assist clinicians in diagnosis and decision making.

**Methods:**

A retrospective MRI dataset of patients diagnosed with CM1 and healthy individuals with normal brain MRIs from the period January 2010 to May 2020 was used to train ResNet50 and VGG19 CNN models to automatically classify images as CM1 or normal. A total of 101 patients diagnosed with CM1 requiring surgery and 111 patients with normal brain MRIs were included (median age 30 with an interquartile range of 23–43; 81 women with CM1). Isotropic volume transformation, image cropping, skull stripping, and data augmentation were employed to optimize model accuracy. K-fold cross validation was used to calculate sensitivity, specificity, and the area under receiver operating characteristic curve (AUC) for model evaluation.

**Results:**

The VGG19 model with data augmentation achieved a sensitivity of 97.1% and a specificity of 97.4% with an AUC of 0.99. The ResNet50 model achieved a sensitivity of 94.0% and a specificity of 94.4% with an AUC of 0.98.

**Conclusions:**

VGG19 and ResNet50 CNN models can be trained to automatically detect clinically significant CM1 on MRI with a high sensitivity and specificity. These models have the potential to be developed into clinical support tools in diagnosing CM1.

**Supplementary Information:**

The online version contains supplementary material available at 10.1007/s00234-022-02921-0.

## Introduction

Chiari malformations are a group of disorders characterized by anatomical abnormalities of the craniocervical junction (CCJ) with involvement of the cerebellum and brainstem. Chiari malformation type 1 (CM1) is the most common Chiari malformation [1–3]. The precise frequency of CM1 is not known; however, since the advent of MRI, CM1 has been increasingly detected with some studies estimating a prevalence of 1–3.6% [4, 5]. The precise natural history of CM1 has not been established and management is generally indicated for patients with severe symptoms, progressive neurological deficits, or those affected by significant syringomyelia with the goal of alleviating symptoms and preventing neurological deterioration [6]. Diagnosis of CM1 rests on identification of displacement of the cerebellar tonsils below the foramen magnum [7]. Currently, it is most widely accepted that a herniation of 5 mm or more is the minimum criterion for diagnosis of CM1 [8–10]. More recently, there has also been recognition of intermediate Chiari subtypes with clinical entities such as the Chiari 0 malformation that is not associated with any cerebellar tonsillar herniation, challenging the widely used 5 mm cutoff for CM1 [11–15].There are other radiological findings that are known to be associated with CM1 such as syringomyelia, hydrocephalus, and skeletal anomalies such as platybasia and a hypoplastic posterior cranial fossa[16–18]. As there are no other tissue or blood biomarkers for CM1, diagnosis currently relies on neuroimaging primarily through MRI and identification of the known associated neuroanatomical anomalies.

Artificial Intelligence (AI) techniques have been applied to assist and improve the diagnosis of a number of pathologies across a range of anatomical regions[19] with promising results, especially with regards to pathologies of neurosurgical interest [20]. In particular, the use of machine learning techniques in the application of image-recognition tasks in radiology is a promising emerging technology with the potential to drastically improve the efficiency and ability of radiology in the clinical setting [19]. In recent years, deep learning, an AI technique utilizing convolutional neural networks (CNN), has been shown to yield high sensitivity in automated detection of pathology on medical imaging with a similar (or even higher) performance to medical experts. A supervised learning approach is typically used to train CNNs where end-to-end labelled data is input into the network including cases with a known diagnosis.

Deep learning algorithms and CNNs have been applied in research for radiological diagnosis of a number of pathologies [21–26]; however, current literature on AI applications in CM1 is limited. Urbizu et al. [27, 28] proposed a diagnostic predictive model based on machine learning, identifying and utilizing different anatomical morphometric parameters rather than the standard sole use of the measurement of tonsillar herniation. This is further evidence of the presence of other anatomical factors that could be important in the neuroradiological diagnosis of CM1 which may be too subtle for human detection but could be resolved by means of an AI-based data mining approach.

To our knowledge, there has not been a study applying a deep learning methodology in the automated diagnosis of CM1 in the literature and the present study aims to develop on this with the ultimate goal of expanding the use of AI in CM1 research and clinical applications. A supervised learning approach was utilized with end-to-end labelled MRI images used to train and subsequently test two CNNs. We also tested the effectiveness of different modifications to the CNN and input data for the optimization of the diagnostic performance. The use of a CNN in the analysis and automated diagnosis of CM1 can open the doors to developing AI-assisted clinical decision tools for controversial areas in the clinical management of CM1 such as management of oligosymptomatic patients, optimization of the surgical technique, and prediction of the clinical outcome and potential for post-operative recurrence.

## Methods

### Study participants

A total of 101 patients who had a diagnosis of CM1 from January 2010 to May 2020 with electronically accessible imaging and who underwent primary surgical intervention at our institution were retrospectively identified; a cohort of 111 patients with normal brain MRIs was also retrieved. Patients had been diagnosed and treated by practicing neurosurgeons and radiologists and as such there were no patients included with possible alternate diagnoses such a spontaneous intracranial hypotension. Normal brain MRIs were defined as individuals with no radiological intracranial pathology as confirmed by certified radiologists (at least 5 years or more experience). To match the CM1 cohort with the normal participants’ cohort, the T1 fluid-attenuated inversion recovery (FLAIR) sequence in the sagittal plane was identified as the most consistently obtainable sequence between the two groups. Matching the sequences between the two groups maximized consistency of the resolutions of the images avoiding bias in the training of the CNN models.

Images were acquired on a 3 T MR unit (Siemens Magnetom Verio, Erlangen, Germany) with the following parameters: TR/TE/TI 2000/8.4/800 ms; flip angle 150°; field-of-view 240 × 240 mm^2^, slice thickness 4.5 mm; matrix 320 × 320. All imaging data were saved to secure private servers for the computational analysis. Since 2010, our institute has obtained consent from all patients undergoing treatment for clinical research using their medical data approved by a clinical human research ethics committee. All participants included in this study had signed this consent. This study was approved by the University Human Research Ethics Committee.

### MRI data pre-processing

**Fig. 1:**
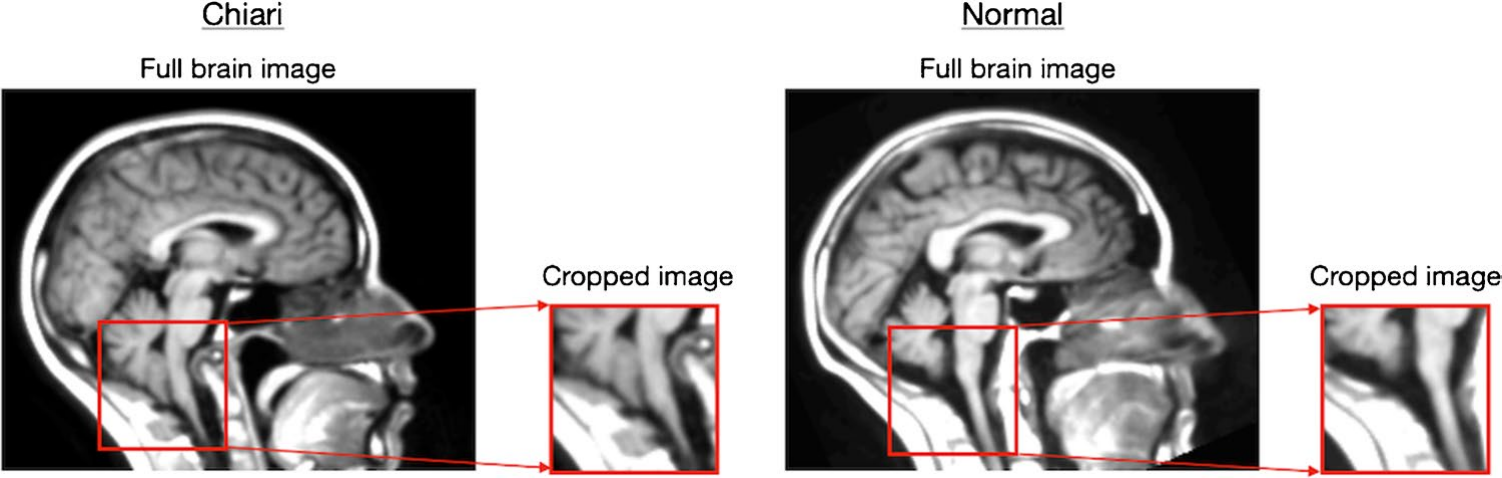
Examples of an image cropped to 64 × 64 pixels of the craniocervical junction without skull stripping

All MRI volumes were anonymized and transformed to isotropic volumes (1 mm^3^ voxel size) to homogenize the different spatial resolutions and orientations of the images as the images were acquired from multiple sites. Following the isotropic transformation step, volumes were aligned to a brain atlas using the Insight Toolkit (ITK) library. First, the ITK Multi-resolution Image Registration Method with Multi-resolution Pyramid Image Filter and Versor Rigid 3D Transform Optimizer was used to estimate the rotation, scaling, and offset parameters. Then the ITK Centered Transform was applied using only rotation and offset parameters obtained from the Versor Rigid 3D Transform [29]. The co-registration to a brain atlas allowed for automatic selection of a single-central sagittal slice of each initial volume to be used in the CNN models (Fig. 1).

### Image datasets

**Fig. 2:**
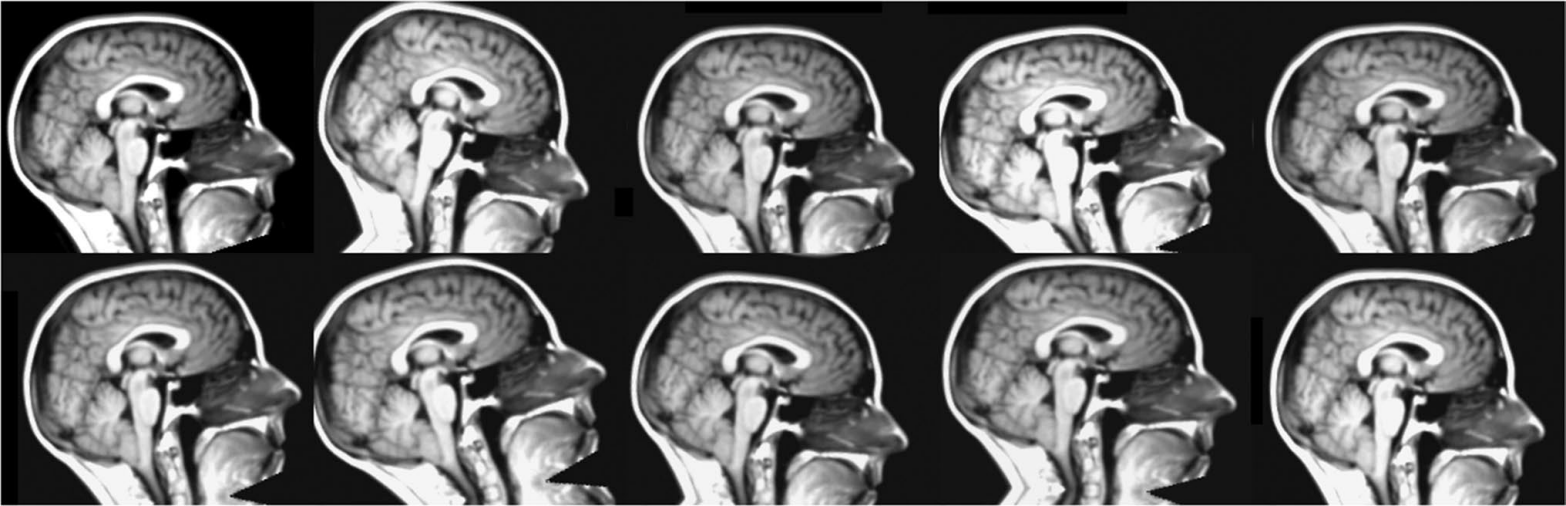
Examples of images following data augmentation in the starting dataset (original image is in the top left corner)

Four types of image dataset were compared to determine the best image format for the training of the neural networks. The training and testing dataset consisted of full images, cropped images, skull stripped full images, and skull stripped cropped images. The full images were the whole single sagittal images automatically selected after the application of the pre-processing pipeline. The cropped images contained an automatically selected 64 × 64 pixel section of the region of interest (coordinates mapped to the reference atlas volume) at the CCJ where herniation of the cerebellum into the foramen magnum occurs (Fig. 2). The CCJ region was selected automatically as the same image coordinates were utilized for the whole dataset after registration of the MRI volumes to the reference atlas volume. Skull stripped images were derived using the ITK filter “StripTsImageFilter” [30, 31]. All images obtained for all four datasets were normalized to have the same pixel intensity value average as in the ImageNet dataset, thus allowing the use of pre-trained weights in CNN models.

### Deep convolutional neural network models

The models used in this study were ResNet50 and VGG19, the most used CNN models in the biomedical sciences. Both models were initialized with ImageNet pre-trained weights, thus enabling transfer learning, i.e., a model that must be trained on different datasets is “transferred” to a different problem and set of information. This was particularly useful given the small dataset available as transfer learning is proven to speed-up the training steps and improve the accuracy. Since the Chiari image dataset is very different from the ImageNet dataset (that largely consists of images of everyday scenes and not medical imaging), all the layers were trainable. In this way, the initial weights were used to extract the low-level features in the first layers whilst allowing the layers to be updated to better match low-level features of the MRI images, especially in consideration of the fact that no color information is contained in grey scale MRI images.

Each model was trained using the 135 training images and 34 validation images, in one image format selected from the four dataset types that had been derived (i.e., non-skull stripped full image, non-skull stripped cropped image, skull stripped full images, and skull stripped cropped images). The number of epochs for each training process was 1000. Categorical cross entropy was used for the loss function. Only the best model weights were saved after each training epoch based on the value of the validation loss. Batch normalization and a batch size of 16 were used to help reducing over-fitting as well as high dropout rates (0.5 and 0.8) on the last dense layer. The hyperparameters of the training process were fine-tuned through experimentation and it was found that an Adam optimizer with a learning rate of 1e-5 and a weights decay of 1e-6 gave better results.

### Data augmentation

To improve the training process with a relatively small number of images, the training dataset was augmented by randomly shifting, zooming, rotating, and shearing the initial images. Brightness was also randomly changed on the original training dataset. The parameters used for the data augmentation were selected to emulate more positions and shapes of the cerebellar tonsillar herniation without an excess of deformation. To achieve this, a random rotation range of 15° clockwise and anticlockwise was performed, 10 pixels for horizontal and vertical random shift, a zoom range of 5%, shear range of 1.2, and a brightness range from 75 to 130% of the original pixel intensity value. Figure 2 shows some examples of augmented images starting from one sample.

### K-fold cross validation

**Fig. 3:**
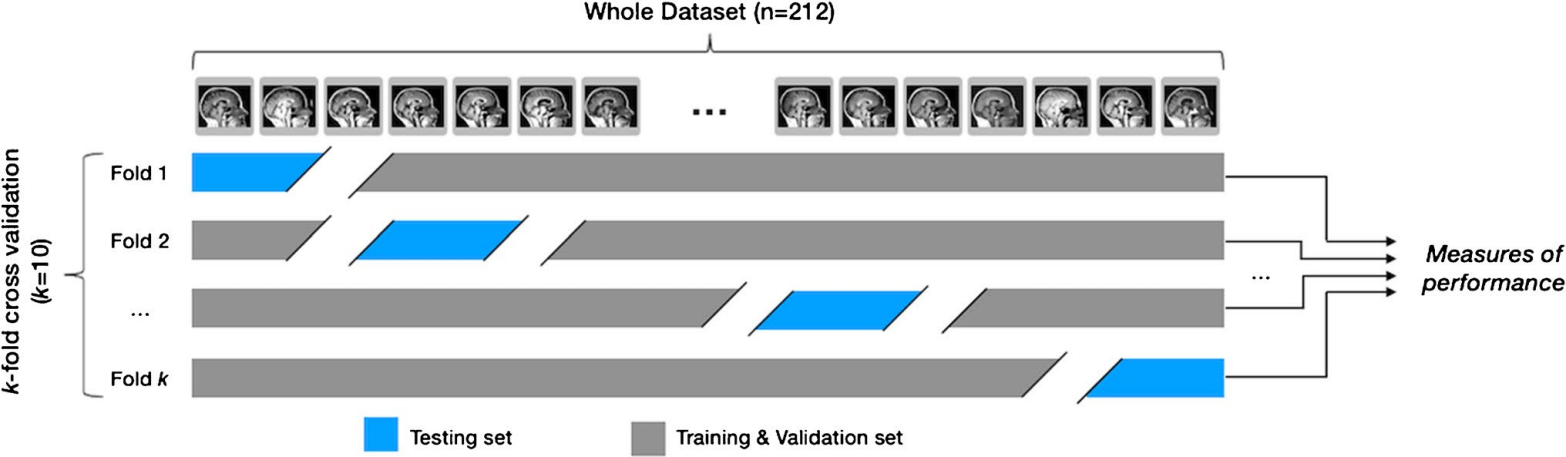
Graphical representation of the k-fold cross validation technique (*k* = 10 in this study)

To evaluate the performance of the deep learning models, a testing dataset is randomly selected from the whole dataset and held out for evaluation of the performance of the model after it has been trained. Given the small sample size of our dataset, precision of the evaluation of the final accuracy of each model was prone to random bias. Small changes in the measured accuracy were mainly due to the random split of the training and testing dataset. Therefore, the *k*-fold cross validation method was used to estimate the precision of the model’s performance where *k* is an arbitrary number of random partitionings of the initial dataset (*k* = 10 in this study). Within each fold, the dataset was split randomly into the training, validation, and testing subsets, i.e., 43 participants (20% of the dataset) were held out as a testing dataset; of the remaining 169 participants, 135 (64% of the dataset) were used for the training of the CNNs and 34 (16% of the dataset) for validation. A graphical representation of this is seen in Fig. 3. Since the split was random, the ratio between Chiari and Normal participants used for the training was not constant but still balanced for a binary classification task (ranging from a minimum of 56 Chiari cases with 79 normal participants to a maximum of 65 Chiari participants with 70 normal participants).

## Results

Table 1 shows the characteristics of the 101 participants that were included in this study. There was a preponderance of female participants (80.2%) which is not unexpected given the well-established increased prevalence of adult CM1 in females[32]. The age of the participants spanned from 5 to 66 years with a median of 30 years (interquartile range from 23–43 years). The age range of the normal participants was from 4 to 93 years (interquartile range from 29–66 years) with 63 females (56.8%) and 48 males (43.2%). The mean cerebellar ectopia was 10.7 mm with a range extending from 4 to 24 mm. The patient with 4 mm of ectopia had a band of dura at the CCJ attaching to the cerebellar tonsils with evidence of CSF flow dysfunction at the CCJ on CSF flow studies and characteristic clinical features suggestive of a diagnosis of a CM1 given the underlying pathophysiology. The inclusion of this patient allows for recognition of a spectrum of cerebellar tonsillar herniation in Chiari malformations in the CNN models.

**Table 1:** Patient characteristics and presenting symptoms

Characteristic	Value (*N* = 101)
**Age (years)**	
Median (interquartile range)	30 (23–43)
Range	5–66
**Gender (%)**	
Female	81 (80.2)
Male	20 (19.8)
**Cerebellar ectopia (mm)**	
Mean (range)	10.7 (4–24)
**Syringomyelia (%)**	
Present	36 (35.6)
Absent	65 (64.4)
**Symptoms (%)**	
Headache	86 (85.1)
Limb pain	20 (19.8)
Hypoesthesia	17 (16.8)
Paresthesia	38 (37.6)
Limb weakness	10 (9.9)
Vertigo	20 (19.8)
Nystagmus	4 (4.0)

Table 2 shows the initial testing of the CNN models that were used in this study with the corresponding dataset types. The ResNet50 model with a dropout rate of 0.5 achieved sensitivities ranging from 81.8 to 86.4% and specificities ranging from 71.4 to 95.2% (AUC 0.89–0.97). The VGG19 model with a dropout rate of 0.5 achieved sensitivities ranging from 81.8 to 86.4% and specificities ranging from 77.3 to 86.4% (AUC 0.94–0.98). Amongst the two models, the cropped (64 × 64 pixel section of the CCJ) and non-skull stripped datasets were found to have the highest sensitivities and specificities. From this initial testing of different combinations of CNN models and starting image datasets, the VGG19 model with non-skull stripped and cropped dataset images set to a dropout rate of 0.8 was found to achieve the highest sensitivity and specificity of 95.5% and 100% respectively (AUC 1.0).

**Table 2:** Measured accuracy of different combinations of convolutional neural networks and dataset settings

CNN model	Sensitivity (%)	Specificity (%)	AUC
**ResNet 50**			
**Dropout rate 0.5**			
Non-cropped images			
Non-skull stripped	86.4%	71.4%	0.89
Skull stripped	81.8%	90.5%	0.93
64 × 64 pixel cropped images			
Non-skull stripped	86.4%	95.2%	0.98
Skull stripped	81.8%	95.2%	0.96
**VGG19**			
**Dropout rate 0.5**			
Non-cropped images			
Non-skull stripped	86.4%	90.5%	0.97
Skull stripped	77.3%	100%	0.98
64 × 64 pixel cropped images			
Non-skull stripped	86.4%	100%	1.00
Skull stripped	77.3%	100%	0.94
**Dropout rate 0.8**			
Non-cropped images			
Non-skull stripped	81.8%	100%	1.00
Skull stripped	77.3%	100%	0.97
64 × 64 pixel cropped images			
Non-skull stripped	95.5%	100%	1.00
Skull stripped	72.7%	100%	0.95

*AUC* area under receiver operating characteristic curve

The tenfold cross validation performed on the VGG19 model with the non-skull stripped and cropped dataset images achieved a sensitivity of 98.4% and a specificity of 94.8% (AUC 0.99) (Table 3). Similarly, the calculated sensitivity and specificity for the ResNet 50 model (also with a dropout rate of 0.8) from tenfold cross validation was 81.2% and 93.1% respectively (AUC 0.94).

**Table 3:** Comparison between ResNet50 and VGG19 with and without data augmentation (average results using tenfold cross validation)

Model	Data Augmentation	Tenfold validation	Tenfold testing	Sensitivity	Specificity	AUC
VGG19	No	95.3%	96.5%	98.4%	94.8%	99.3%
VGG19	Yes	97.4%	97.2%	97.1%	97.4%	99.2%
ResNet50	No	93.2%	86.7%	81.2%	93.1%	94.1%
ResNet50	Yes	92.6%	94.0%	94.0%	94.4%	98.3%

*AUC* area under receiver operating characteristic curve

The performance of both models was largely improved by data augmentation. Table 3 shows the tenfold cross validations results on validation and testing dataset for both models with and without data augmentation (using the cropped and non-skull stripped datasets) and Table 4 shows a tenfold cross validation run for VGG19 with optimized pre-processing data settings. Whilst the specificity of the VGG19 model improved with data augmentation from 94.8% to 97.4%, the sensitivity decreased slightly from 98.4 to 97.1% (AUC 0.99). Both the sensitivity and specificity of the ResNet 50 model improved to 94.0 and 94.4% respectively (AUC 0.98).

**Table 4:** Ten-fold cross validation values for CNN model VGG19 with 64 × 64 cropped, non-skull stripped datasets with data augmentation achieving the highest accuracy in testing

Run	Validation	Testing	Sensitivity	Specificity	AUC
1	97.1%	97.7%	100%	94.0%	1.00
2	97.1%	100.0%	100%	100%	1.00
3	97.1%	97.7%	100%	96.0%	0.98
4	97.1%	95.3%	100%	94.0%	1.00
5	94.1%	95.3%	89.0%	100%	1.00
6	100.0%	100.0%	100%	100%	1.00
7	100.0%	97.7%	96.0%	100%	1.00
8	97.1%	100.0%	100%	100%	1.00
9	94.1%	95.3%	96.0%	95.0%	1.00
10	100.0%	93.0%	90.0%	96.0%	0.94

*AUC* area under receiver operating characteristic curve

## Discussion

The application of deep learning CNNs in neuroradiology is an expanding area of research; however its validation in the automated diagnosis of neuroanatomical disorders such as CM1 is limited. This study aimed to apply and validate CNN models in automated diagnosis and identification of clinically significant CM1 to demonstrate the utility of deep learning in developing decision-making support tools for clinicians. We trained VGG19 and ResNet50 CNN models to diagnose CM1 on MRI (utilizing sagittal T1 FLAIR sequences) achieving a high level of sensitivity and specificity as validated with a tenfold cross validation. The CNN models were optimized by utilizing pre-processing image modification techniques including isotropic transformation, region of interest cropping, and data augmentation. The VGG19 model produced the best results with a sensitivity of 97.1% and specificity of 97.4% achieved when data augmentation was used (AUC 0.99).

There are limitations in this study that should be considered with the interpretation of the results. The dataset of 212 participants was small and limited to a single center, which limited the case numbers that were usable in the training, testing, and validation subsets and thus performing unsupervised learning to look for clustering of neuroanatomical features was unable to be performed. The small dataset size also prevented an analysis of unilateral or asymmetrical CM1 as the inclusion of stacks of images lateral to the midline sagittal image in the pre-processing pipeline would introduce a degree of variability necessitating a larger number of participants. The random splitting of the data for the cross validation also represented a potential source of random error; however, this was circumvented by use of the tenfold cross validation method with data augmentation. Thus, the final sensitivity and specificity rates achieved by the CNN models in this study were still high (97.2% for VGG19).

This study was also limited by the MRI sequence availability as we were restricted to using sagittal T1 MRI sequences for consistency between both the CM1 patient dataset and the normal brain dataset. This is partly due to the small sample size necessitating a more uniformly consistent dataset to minimize any bias in the training of the CNN models. Amongst the datasets collected for this study, the T1 sagittal FLAIR sequence was most consistently available between CM1 and normal participants. Although an isotropic transformation was applied to the image datasets, the use of a single type of MRI sequence and anatomical plane represents a further restriction to the generalizability of this study.

The highest sensitivity and specificity were achieved in the VGG 19 model with the 64 × 64 cropped and non-skull stripped data augmented images (Table 2). Skull stripping with the ITK filter was found to crop out pertinent features at the CCJ (such as the tonsillar herniation) and hence demonstrated inferior performance. The original rationale for testing skull-stripped images was to further enhance the accuracy of the models by eliminating regions of the head that are far removed from the region of interest. Skull stripping with the ITK filter was found to also crop out pertinent features at the CCJ (such as the tonsillar herniation) and hence demonstrated inferior performance. This is not surprising as it known that anatomical differences in the skull base or cranial vault are known to be important factors in the pathogenesis of CM1 [33, 34]. This highlights a need for new techniques that can incorporate the spinal content with the exclusion of the surrounding bone to improve the currently available skull stripping methodologies.

**Fig. 4:**
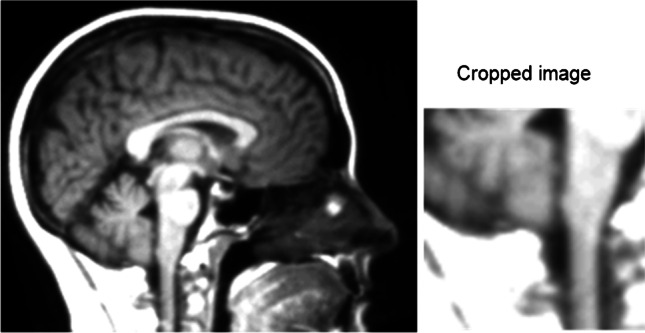
Example of a normal MRI misclassified as Chiari

**Fig. 5:**
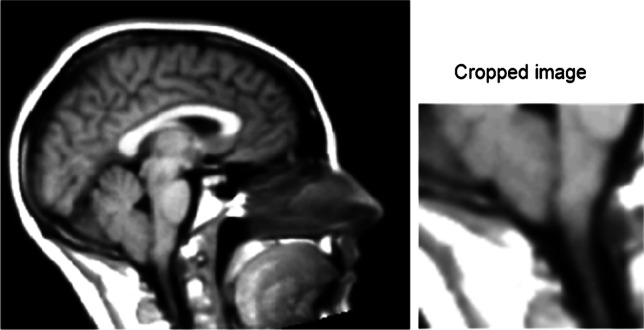
Example of a Chiari MRI misclassified as normal

A review of the misclassified cases revealed that they were borderline cases of CM1 with evidence of crowding at the CCJ without overt tonsillar herniation on MRI. Figure 4 shows an example of a normal patient MRI misclassified as CM1 by the models. Although there is no significant tonsillar herniation seen, there is suggestion of cerebellar crowding at the CCJ which we suspect is the reason for the misclassification. Similarly, in Fig. 5, a CM1 patient was misclassified as normal with a similar appearance to the posterior fossa with no clear tonsillar herniation. In this case, a combination of the clinical history and review of the T2 sequence is what lead to the diagnosis of CM. T2 sequences allow for better delineation of cerebellar tonsillar herniation and posterior fossa crowding and would have been the preferred MRI sequence in this study although this sequence was not readily available between the CM1 and control groups. It is not surprising that the CNN model misclassified this case in the absence of clinical data given the equivocal appearance of the posterior fossa on the T1 sequence. The fact that the misclassified cases were borderline lends weight to the validity of the trained CNN model as the misclassifications in the models only arose in truly equivocal cases.

The results of this study represent the first step towards development of an AI model that can assist clinicians in recognizing clinically significant CM1 and has potential to be developed into a tool for diagnosis, surgical outcome prognostication, developing optimal surgical techniques, and guiding management of recurrence. Clinical studies on these problems are difficult to execute and there are no highly powered studies to guide clinicians. The current VGG19 model could be augmented by incorporating clinical outcome data, post-operative imaging, and post-operative outcomes that are matched to the input images to create a multifaceted clinical support tool. This data can be collated and unsupervised learning analyses using the models can be used to identify clustering of pertinent radiological and clinical features to further elucidate the underlying pathophysiology of disrupted cerebrospinal fluid circulation in CM1. The diagnostic scope of this model could also be further enhanced by including datasets of other structural pathological entities to train the CNN to allow automated diagnosis of a range of other neuroanatomical anomalies.

## Conclusion

In conclusion, the high sensitivity and specificity rates achieved in these CNN models represents a successful approach to automated recognition of clinically significant CM1 in MRI images. Whilst there are some limitations to the generalizability of the study, these results represent a starting point for further AI assisted research of CM1. Further studies including larger sample sizes, post-operative imaging, and clinical data are needed to improve the accuracy and generalizability of the models and to work towards developing a clinical support tool for managing CM1.

## Funding infromation

The authors received no financial support for the research and authorship of this article.

## Supplementary Information

Below is the link to the electronic supplementary material.

Supplementary file1 (DOCX 41 KB)

## Abbreviations

CMI: Chiari type I malformation
MRI: Magnetic resonance imaging
CNN: Convolutional neural network
